# Prognosis Score System to Predict Survival for COVID-19 Cases: a Korean Nationwide Cohort Study

**DOI:** 10.2196/26257

**Published:** 2021-02-22

**Authors:** Sung-Yeon Cho, Sung-Soo Park, Min-Kyu Song, Young Yi Bae, Dong-Gun Lee, Dong-Wook Kim

**Affiliations:** 1 Catholic Hematology Hospital, College of Medicine The Catholic University of Korea Seoul Republic of Korea; 2 Division of Infectious Diseases, Department of Internal Medicine, College of Medicine The Catholic University of Korea Seoul Republic of Korea; 3 Division of Hematology, Department of Internal Medicine, College of Medicine The Catholic University of Korea Seoul Republic of Korea; 4 Data Research Institute YMDtech Inc Seoul Republic of Korea; 5 St. Mary's Gong-Gam Mental Health Clinic, Siheung-si Gyeonggi-do Republic of Korea

**Keywords:** COVID-19, length of stay, mortality, prognosis, triage, digital health, prediction, cohort, risk, allocation, disease management, intensive care, decision making

## Abstract

**Background:**

As the COVID-19 pandemic continues, an initial risk-adapted allocation is crucial for managing medical resources and providing intensive care.

**Objective:**

In this study, we aimed to identify factors that predict the overall survival rate for COVID-19 cases and develop a COVID-19 prognosis score (COPS) system based on these factors. In addition, disease severity and the length of hospital stay for patients with COVID-19 were analyzed.

**Methods:**

We retrospectively analyzed a nationwide cohort of laboratory-confirmed COVID-19 cases between January and April 2020 in Korea. The cohort was split randomly into a development cohort and a validation cohort with a 2:1 ratio. In the development cohort (n=3729), we tried to identify factors associated with overall survival and develop a scoring system to predict the overall survival rate by using parameters identified by the Cox proportional hazard regression model with bootstrapping methods. In the validation cohort (n=1865), we evaluated the prediction accuracy using the area under the receiver operating characteristic curve. The score of each variable in the COPS system was rounded off following the log-scaled conversion of the adjusted hazard ratio.

**Results:**

Among the 5594 patients included in this analysis, 234 (4.2%) died after receiving a COVID-19 diagnosis. In the development cohort, six parameters were significantly related to poor overall survival: older age, dementia, chronic renal failure, dyspnea, mental disturbance, and absolute lymphocyte count <1000/mm^3^. The following risk groups were formed: low-risk (score 0-2), intermediate-risk (score 3), high-risk (score 4), and very high-risk (score 5-7) groups. The COPS system yielded an area under the curve value of 0.918 for predicting the 14-day survival rate and 0.896 for predicting the 28-day survival rate in the validation cohort. Using the COPS system, 28-day survival rates were discriminatively estimated at 99.8%, 95.4%, 82.3%, and 55.1% in the low-risk, intermediate-risk, high-risk, and very high-risk groups, respectively, of the total cohort (*P*<.001). The length of hospital stay and disease severity were directly associated with overall survival (*P*<.001), and the hospital stay duration was significantly longer among survivors (mean 26.1, SD 10.7 days) than among nonsurvivors (mean 15.6, SD 13.3 days).

**Conclusions:**

The newly developed predictive COPS system may assist in making risk-adapted decisions for the allocation of medical resources, including intensive care, during the COVID-19 pandemic.

## Introduction

Since the outbreak of unexplained pneumonia in Wuhan, China, in December 2019, which was subsequently identified as COVID-19 caused by the newly discovered pathogen SARS-CoV-2, the COVID-19 pandemic remains active in over 180 countries [1,2]. Globally, as of November 6, 2020, 1,231,017 of a total of 48,534,508 patients with COVID-19 have died, representing an overall infection fatality rate of 2.54% [3,4]. The clinical spectrum of COVID-19 includes asymptomatic or presymptomatic, upper and lower respiratory tract infections and acute respiratory distress syndrome [5-8]. Although the majority of viral infections are self-limiting, COVID-19 cases that are severe (ie, dyspnea, hypoxia, or >50% of lung involvement observed on imaging within 24–48 h) or critical (ie, respiratory failure, shock, or multiple organ failure) are of global concern and require medical resources for intensive care. The proportion of severe or critical COVID-19 cases and the corresponding case fatality rates vary by region and country, ranging from 10%-30% [8-10] to 2%-10%, respectively [3,4].

Most severe or critical cases occur in older patients or those with underlying comorbidities such as cancer, chronic obstructive pulmonary diseases, heart failure, or diabetes [11-13]. The clinical course of patients with COVID-19 also depends on multiple factors, including the immune status of the host, viral load of SARS-CoV-2, genetic diversity of the virus, and underlying diseases. However, details of viral factors and the host immune status (eg, cytokines released) are difficult to analyze in a real-world setting. Therefore, prognosis prediction systems should comprise basic factors such as initial symptoms at diagnosis, vital signs, hemogram parameters, and major underlying comorbidities.

As the global pandemic continues, the ability to detect, in a timely manner, patients with COVID-19 who are at a high risk of death and provide them with intensive care is important. Accordingly, the assessment of disease severity or mortality probability can be used to establish a sustainable strategy. Therefore, in this study, we aimed to develop an easy and simple scoring system that can predict COVID-19 mortality according to the patient’s initial presentation and several major underlying comorbidities. Additionally, we investigated the association between the length of hospital stay and disease severity and survival status of patients with COVID-19.

## Methods

### Study Design and Data Source

This was a nationwide, retrospective cohort study on COVID-19 cases in Korea. For the purpose of this study, COVID-19 cases were defined based on laboratory confirmation of infection and positive results of SARS-CoV-2 reverse transcription polymerase chain reaction assays performed using the testing kits approved by the Korea Ministry of Food and Drug Safety, irrespective of the patient’s clinical signs and symptoms [14,15]. Clinical data of patients in this cohort were managed by the Korea Disease Control and Prevention Agency (KDCA) and disclosed to the researchers after application and consent for research purposes in July 2020. The clinical and epidemiological information thus obtained included data of 5628 COVID-19 cases collected between January and April 2020. KDCA is an organization that aims to protect the Korean population from diseases, including emerging infectious diseases such as COVID-19, through national surveillance, health care research, and promotion of policies regarding disease prevention management. Patient data collected for this study included demographic and epidemiological characteristics, hemogram parameters at admission, maximal severity, and clinical outcome obtained from designated hospitals. Patients in the final cohort were randomly allocated to two subcohorts by using a random number generator: two-thirds into the “development cohort” and the remaining one-third into the “validation cohort.” The predictive score was developed based on the development cohort, whereas the power of prediction was explored in the validation cohort.

This study was approved by the institutional review board of Seoul St. Mary’s Hospital, Seoul, Korea (KC20ZADI0654). Individual patient consent was waived because the data retrieved were anonymous and publicly available.

### COVID-19 Management Setting

In Korea, all suspected or confirmed cases of COVID-19 must be reported to the KDCA, as COVID-19 is regarded as a notifiable infectious disease. As a result, all patients with laboratory-confirmed COVID-19 were admitted to designated hospitals or residential treatment centers for isolation, monitoring of symptoms, and treatment. Clinical severity was classified into the following 8 levels according to patient performance, oxygen requirement, and organ failure [16]: (1) no limit of activity, (2) limited activity without oxygen supplementation, (3) requirement of oxygen supply with nasal cannula, (4) requirement of oxygen supply with facial mask, (5) requirement of noninvasive ventilation, (6) requirement of invasive ventilation, (7) requirement of extracorporeal membrane oxygenation (ECMO) for multiple organ failure, or (8) death. Death outcomes were evaluated regardless of maximum severity. Patients that required oxygen supply with invasive ventilation or ECMO were considered as invasive intensive care cases.

### Statistical Analysis

Normally distributed numerical variables are presented as mean and SD values. Categorical variables are shown as absolute numbers and their proportions (n, %). The hospital stay durations between two independent groups were compared using Student *t* test. Overall survival was defined as the time from COVID-19 diagnosis to death due to any cause or up to the date of the last follow-up. Death events were censored at the time of hospital discharge for patients who were discharged. Overall survival rates at 14 days and 28 days were calculated using the Kaplan-Meier method and compared using the Log-rank test.

Within the development cohort, all risk factors with a *P* value <.05 in the univariable analysis were entered into the multivariable model to identify factors associated with overall survival. Multivariable analysis was performed using the Cox proportional hazard regression model. We identified potential variables for the final prediction model based on 2000 bootstrap sampled datasets. When a parameter occurred in 60% or more of the bootstrap models, it was evaluated in the final multiple logistics regression model. We then computed the hazard ratios, 95% CIs, and *P* values for all metrics of the bootstrapped datasets in the final regression model. The final parameters used in the scoring system were defined by a *P* value <.05 in the final regression model. To confirm the risk score for each significant parameter, we adjusted the hazard ratio values to a log_e_ scale, followed by the conversion of the respective log_e_ scale to a rounded integer point. In the validation cohort, the area under the receiver operating characteristic (AUROC) curve was measured to evaluate the prediction accuracy of the survival rates after 14 and 28 days. An AUROC value above 0.8 was considered reliable. Among the developed risk groups, we compared the length of hospital stay using one-way analysis of variance. For all statistical analyses, we used R statistical software (ver. 3.6.1, R Foundation for Statistical Computing). Statistical significance was set at *P*<.05.

## Results

### Patient Characteristics

**Figure 1:**
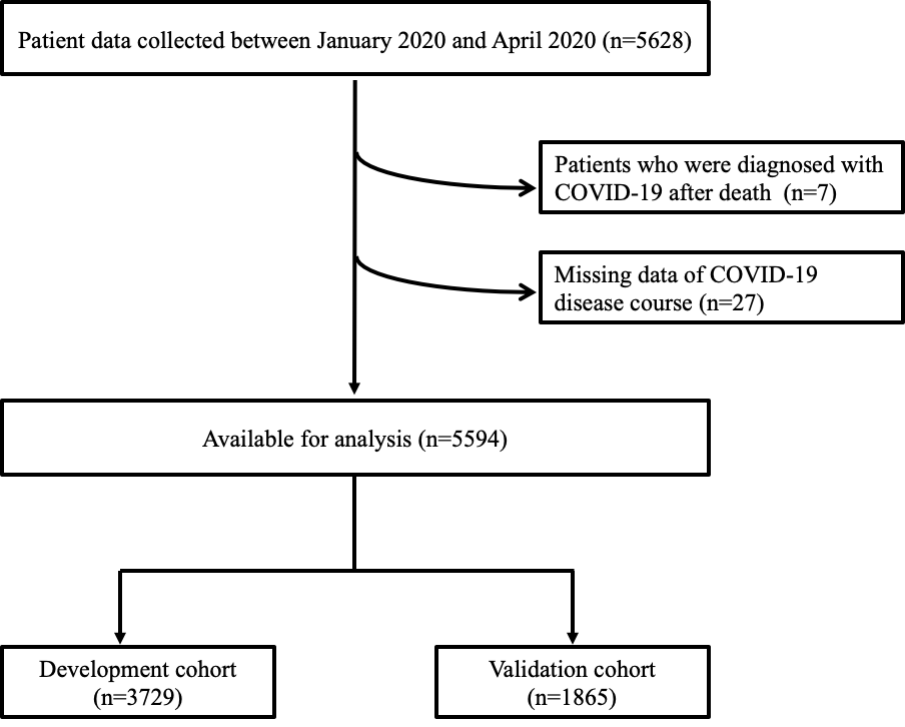
Flow diagram of the nationwide cohort of patients with COVID-19.

In total, 5628 confirmed COVID-19 cases were reported between January and April 2020. Cases with a postmortem diagnosis (n=7) or lack of clinical course data after diagnosis (n=27) were excluded from the analysis. As shown in Figure 1, a total of 5594 patients with COVID-19 were included in this cohort. Overall, 41.2% (2307/5594) of the patients were male, and 52.2% (2919/5594) were aged 50 years or older. Baseline demographics are summarized in Table 1. In the total cohort, the most frequent age distribution was 50-59 years (1140/5594, 20.4%), followed by 20-29 years (1110/5594, 19.8%) and 60-69 years (905/5594, 16.2%). The most frequently reported symptoms included sputum (1610/5594, 28.8%), fever (1300/5594, 23.2%), and dyspnea (662/5594, 11.8%). Common underlying comorbidities reported were hypertension (1196/5594, 21.4%) and diabetes (686/5594, 12.3%). Moreover, 4% (224/5594) of the patients had dementia and 3.2% (179/5594) of them had cardiac diseases. Distribution of these variables between the development (n=3729) and validation (n=1865) cohorts is presented in Table 1.

**Table 1:** Demographics of the study cohorts. All values presented in the table represent data collected at the time of COVID-19 diagnosis.

Variable			Total cohort (N=5594), n (%)	Subcohorts	
				Development cohort (n=3729), n (%)	Validation cohort (n=1865), n (%)
**Demographics, n (%)**					
	**Age (years)**				
		0-9	66 (1.2)	46 (1.2)	20 (1.1)
		10-19	205 (3.7)	146 (3.9)	59 (3.2)
		20-29	1,110 (19.8)	725 (19.4)	385 (20.6)
		30-39	564 (10.1)	378 (10.1)	186 (10)
		40-49	739 (13.2)	487 (13.1)	252 (13.5)
		50-59	1,140 (20.4)	768 (20.6)	372 (19.9)
		60-69	905 (16.2)	605 (16.2)	300 (16.1)
		70-79	542 (9.7)	364 (9.8)	178 (9.5)
		≥ 80	323 (5.8)	210 (5.6)	113 (6.1)
	Gender (male)		2,307 (41.2)	1536 (41.2)	1094 (58.7)
**Comorbidity, n (%)**					
	Hypertension (missing n=3)		1196 (21.4)	795 (21.3)	401 (21.5)
	Diabetes (missing n=3)		686 (12.3)	452 (12.1)	234 (12.5)
	Dementia (missing n=329)		224 (4)	150 (4)	74 (4)
	Cardiac disease (missing n=19)		179 (3.2)	122 (3.3)	57 (3.1)
	Cancer in active treatment^a^ (missing n=4)		143 (2.6)	90 (2.4)	53 (2.8)
	Asthma (missing n=3)		128 (2.3)	82 (2.2)	46 (2.5)
	Chronic hepatic disease^b^ (missing n=326)		82 (1.5)	47 (1.3)	35 (1.9)
	Heart failure (missing n=3)		58 (1)	36 (1)	22 (1.2)
	Chronic renal failure (missing n=3)		55 (1)	36 (1)	19 (1)
	Chronic obstructive lung disease (missing n=3)		40 (0.7)	25 (0.7)	15 (0.8)
	Autoimmune disease (missing n=332)		38 (0.7)	31 (0.8)	7 (0.4)
**Symptoms (missing n=4), n (%)**					
	Sputum		1610 (28.8)	1114 (29.9)	496 (26.6)
	Fever		1300 (23.2)	852 (22.8)	448 (24)
	Dyspnea		662 (11.8)	454 (12.2)	208 (11.2)
	Diarrhea		516 (9.2)	345 (9.3)	171 (9.2)
	Nausea or vomiting		244 (4.4)	168 (4.5)	76 (4.1)
	Fatigue		233 (4.2)	164 (4.4)	69 (3.7)
	Mental disturbance, n (%)		32 (0.6)	22 (0.6)	10 (0.5)
**Systolic blood pressure (mmHg) (missing n=135), n (%)**					
	<120		1306 (23.3)	907 (24.3)	399 (21.4)
	120-129		1138 (20.3)	733 (19.7)	405 (21.7)
	130-139		1084 (19.4)	705 (18.9)	379 (20.3)
	140-159		1418 (25.3)	960 (25.7)	458 (24.6)
	≥160		513 (9.2)	330 (8.8)	183 (9.8)
**Diastolic blood pressure (mmHg) (missing n=135), n (%)**					
	<80		2102 (37.6)	1401 (37.6)	701 (37.6)
	80-89		1797 (32.1)	1201 (32.2)	596 (32)
	90-99		1056 (18.9)	686 (18.4)	370 (19.8)
	≥100		504 (9)	347 (9.3)	157 (8.4)
**Heart rate (per min) (missing n=122), mean (SD)**			85.8 (15.1)	85.8 (15.1)	85.8 (15.1)
	<110, n (%)		5136 (91.8)	3,374 (90.5)	1709 (91.6)
	≥110, n (%)		336 (6)	272 (7.3)	117 (6.3)
**Body temperature (°C) (missing n=37), mean (SD)**			36.9 (0.6)	36.9 (0.6)	36.9 (0.6)
	<38°C, n (%)		5348 (95.6)	3523 (94.5)	1752 (93.9)
	≥38°C, n (%)		209 (3.7)	179 (4.8)	103 (5.5)
**Baseline hemogram**					
	**Hemoglobin (g/dL) (missing n=1519), mean (SD)**		13.3 (1.8)	13.3 (1.7)	13.3 (1.8)
		≥12.5, n (%)	2882 (51.5)	1923 (51.6)	959 (51.4)
		<12.5, n (%)	1193 (21.3)	773 (20.7)	420 (22.5)
	**Absolute lymphocyte count (per mm^3^) (missing n=1542), mean (SD)**		1691 (1,054)	1697 (955)	1,681 (1,225)
		≥1000, n (%)	3266 (58.4)	2161 (58)	1105 (59.2)
		<1000, n (%)	786 (14.1)	518 (13.9)	268 (14.4)
	**Platelet count (per mm^3^) (missing n=1517), mean (SD)**		236,814 (82,846)	238,377 (82,789)	233,760 (82,900)
		≥100,000, n (%)	3986 (71.3)	2634 (70.6)	1352 (72.5)
		<100,000, n (%)	91 (1.6)	62 (1.7)	29 (1.6)
	Follow-up (days), median (95% CI)		25 (24-25)	25 (24-25)	25 (24-25)

^a^Cases that achieved complete cure of cancer were excluded.

^b^Cases with chronic hepatitis were included in this category.

### Clinical Course, Outcome, and Length of Hospital Stay in the Total Cohort

**Figure 2:**
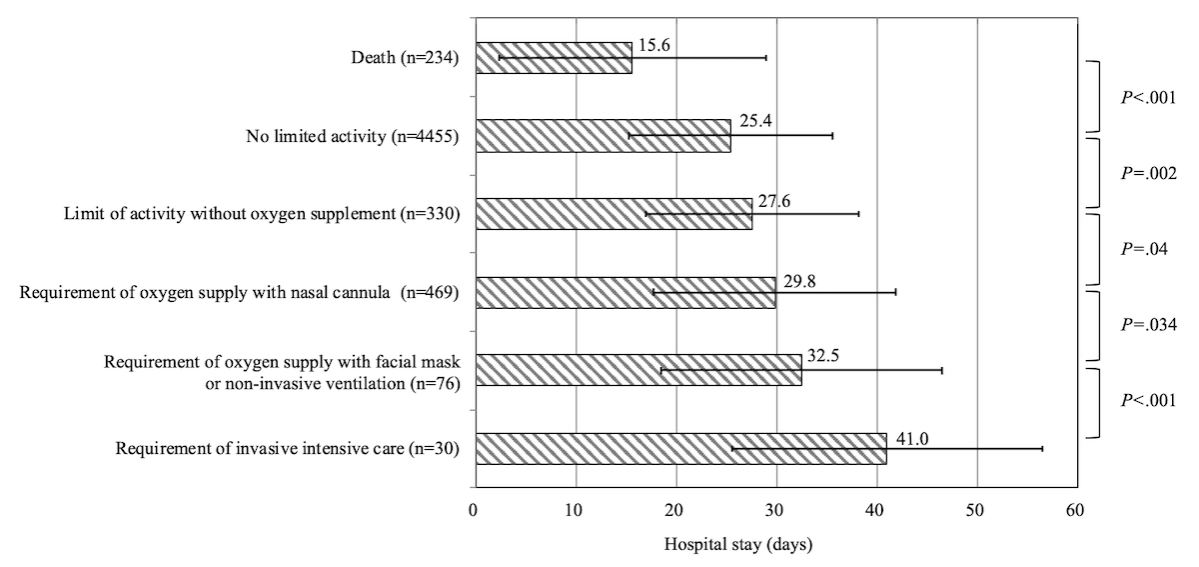
Maximal COVID-19 disease severity and duration of hospital stay. Hospital stay duration differed significantly according to maximal disease severity; the mean length of hospital stay is indicated.

Among the 5594 patients included in the analysis, 234 (4.2%) died after a COVID-19 diagnosis was made, resulting in a cohort case fatality rate of 4.2%. Excluding death, the maximal clinical disease severity during hospitalization was as follows: (1) no limit of activity in 79.6% (4455/5594) of the patients, (2) limited activity without oxygen supplementation in 5.9% (330/5594) of the patients, (3) requirement of oxygen supply with nasal cannula in 8.4% (469/5594) of the patients, (4) requirement of oxygen supply with facial mask or advanced device (such as noninvasive ventilation or high flow oxygen therapy) in 1.4% (76/5594) of the patients, and (5) requirement of invasive intensive care such as invasive ventilation for acute respiratory distress syndrome or ECMO for multiple organ failure in 0.5% (30/5594) of the patients (Figure 2).

Overall, the mean duration of hospital stay was 25.6 (SD 11.0) days. Hospital stay was significantly longer among survivors (mean 26.1, SD 10.7 days) than among nonsurvivors (mean 15.6, SD 13.3 days; *P*<.001). As shown in Figure 2, the higher the severity of clinical course among survivors, the longer was their hospital stay: no limit of activity, mean 25.4 (SD 10.2) days; limited activity without oxygen supplementation, mean 27.6 (SD 10.6) days; oxygen supply with nasal cannula, mean 29.8 (SD 12.0) days; oxygen supply with facial mask or advanced device, mean 32.5 (SD 14.0) days; and invasive intensive care groups, mean 41.0 (SD 15.4) days.

### Analysis of Factors Associated With Overall Survival in the Development Cohort

The univariable analysis identified the following potential factors associated with poor overall survival: age (≥70 or 50-69 years vs <50 years); sex (male); comorbidities such as hypertension, diabetes, dementia, chronic cardiac disease, cancer in active treatment, chronic pulmonary disease, and chronic renal failure; dyspnea, fatigue, mental disturbance, high systolic blood pressure (≥140 mmHg), low diastolic blood pressure (<80 mmHg), tachycardia (heart rate ≥110/min), and fever (≥38°C) at the time of diagnosis; and cytopenia (hemoglobin level <12.5 g/dL, absolute lymphocyte count [ALC] <1000/mm^3^, and platelet count <100,000/mm^3^), as shown in Table 2.

**Table 2:** Univariable analysis for potential factors associated with overall survival in the development cohort. All values presented in the table represent data collected at the time of initial COVID-19 diagnosis.

Factor			Number of patients (n=3729), n (%)		Overall survival rate, % (95% CI)				*P* value			
					At 14 days		At 28 days					
**Age (years)**									<.001			
	<50			1782 (47.8)		99.9 (99.8-100)		99.8 (99.5-100)				
	50-69			1373 (36.8)		98.6 (98-99.2)		98 (97.2-98.8)				
	≥70			574 (15.4)		87 (84.3-89.8)		81.6 (78.3-85)				
**Sex**									.01			
	Female			2193 (58.8)		97.8 (97.2-98.4)		96.8 (96-97.6)				
	Male			1536 (41.2)		96.9 (96-97.8)		95.2 (94.1-96.4)				
**Comorbidity**												
	**Hypertension**									<.001		
		No		2932 (78.6)		98.7 (98.3-99.1)		98 (97.4-98.6)				
		Yes		795 (21.3)		92.7 (90.9-94.6)		89.7 (87.4-91.9)				
	**Diabetes**									<.001		
		No		3275 (87.8)		98.3 (97.8-98.7)		97.3 (96.7-98)				
		Yes		452 (12.1)		91.5 (88.9-94.1)		88.1 (85-91.3)				
	**Dementia**									<.001		
		No		3359 (90.1)		98.3 (97.9-98.7)		97.3 (96.7-97.9)				
		Yes		150 (4)		74.5 (67.8-81.8)		67.4 (60.1-75.6)				
	**Chronic cardiac disease** ^a^									<.001		
		No		3582 (96.1)		97.7 (97.2-98.2)		96.7 (96-97.3)				
		Yes		147 (3.9)		91.1 (86.6-95.8)		85.8 (79.9-92)				
	**Cancer in active treatment**									.03		
		No		3636 (97.5)		97.5 (97-98)		96.3 (95.7-97)				
		Yes		90 (2.4)		95.5 (91.2-99.9)		89.8 (82.5-97.7)				
	**Chronic pulmonary disease** ^b^									<.001		
		No		3628 (97.3)		97.6 (97.1-98.1)		96.5 (95.9-97.2)				
		Yes		101 (2.7)		92 (86.8-97.5)		85.3 (77.9-93.3)				
	**Chronic hepatic disease**									.6		
		No		3463 (92.9)		97.3 (96.7-97.8)		96 (95.2-96.7)				
		Yes		47 (1.3)		97.9 (93.8-100)		95.3 (89.1-100)				
	**Chronic renal failure**									<.001		
		No		3691 (99)		97.5 (97-98)		96.3 (95.7-97)				
		Yes		36 (1)		85.7 (74.9-98.1)		81.2 (68.4-96.4)				
	**Autoimmune disease**									.56		
		No		3476 (93.2)		97.3 (96.7-97.8)		96 (95.3-96.7)				
		Yes		31 (0.8)		96.8 (90.8-100)		90.7 (78.7-100)				
**Symptoms**												
	**Sputum**									.59		
		No		2612 (70)		97.4 (96.8-98.1)		96 (95.2-96.9)				
		Yes		1114 (29.9)		97.4 (96.4-98.3)		96.5 (95.4-97.7)				
	**Dyspnea**									<.001		
		No		3272 (87.7)		98.3 (97.9-98.8)		97.4 (96.8-98)				
		Yes		454 (12.2)		90.9 (88.2-93.6)		87.6 (84.4-90.9)				
	**Diarrhea**									.81		
		No		3381 (90.7)		97.4 (96.8-97.9)		96.1 (95.4-96.8)				
		Yes		345 (9.3)		98 (96.5-99.5)		96.8 (94.8-98.8)				
	**Nausea/vomiting**									.2		
		No		3558 (95.4)		97.5 (97-98.1)		96.3 (95.6-97)				
		Yes		168 (4.5)		95.1 (91.8-98.5)		94.3 (90.8-98)				
	**Fatigue**									.006		
		No		3562 (95.5)		97.5 (97-98.1)		96.4 (95.7-97.1)				
		Yes		164 (4.4)		95 (91.7-98.5)		91.9 (87.6-96.5)				
	**Mental disturbance**									<.001		
		No		3704 (99.3)		97.7 (97.3-98.2)		96.5 (95.9-97.2)				
		Yes		22 (0.6)		45.5 (28.8-71.8)		40.4 (24.2-67.5)				
**Systolic blood pressure (mmHg)**									.02			
	<140			2345 (62.9)		97.8 (97.2-98.4)		96.8 (96-97.6)				
	≥140			1290 (34.6)		96.9 (95.9-97.9)		95.3 (94-96.6)				
**Diastolic blood pressure (mmHg)**									.01			
	<80			1401 (37.6)		96.7 (95.7-97.6)		94.9 (93.7-96.2)				
	≥80			2234 (59.9)		98 (97.4-98.6)		97.1 (96.3-97.8)				
**Heart rate (per min)**									.005			
	<110			3374 (90.5)		97.7 (97.2-98.2)		96.4 (95.7-97.1)				
	≥110			272 (7.3)		94.4 (91.6-97.2)		93.5 (90.6-96.6)				
**Body temperature (°C)**									<.001			
	<38			3523 (94.5)		97.8 (97.3-98.3)		96.4 (95.8-97.1)				
	≥38			179 (4.8)		93.2 (89.6-97)		93.2 (89.6-97)				
**Baseline hemogram**												
	**Hemoglobin (g/dL)**									<.001		
	≥12.5			1923 (51.6)		97.9 (97.2-98.5)		96.8 (95.9-97.7)				
	<12.5			773 (20.7)		93.6 (91.8-95.3)		90.8 (88.7-93.1)				
	**Absolute lymphocyte count (per mm** ^3^ **)**									<.001		
	≥1000			2161 (58)		98.3 (97.8-98.9)		97.7 (97-98.4)				
	<1000			518 (13.9)		89.7 (87.1-92.4)		84.3 (81-87.8)				
	**Platelet count (per mm** ^3^ **)**									<.001		
	≥100,000			2634 (70.6)		96.9 (96.2-97.5)		95.2 (94.3-96.1)				
	<100,000			62 (1.7)		85.3 (76.9-94.7)		83.5 (74.7-93.4)				

^a^Chronic cardiac disease was a composite variable including heart failure and cardiac disease.

^b^Chronic pulmonary disease was a composite variable including asthma and chronic obstructive lung disease.

### COVID-19 Prognosis Score for Predicting Overall Survival

In the bootstrap analysis, we identified that older age (50-69 or ≥70 years) and comorbidities, including dementia, chronic renal failure, presentation of dyspnea, mental disturbance at diagnosis, and ALC <1,000 /mm^3^ were significantly associated with poor overall survival. Assigned risk scores obtained by rounding the log_e_ scale of hazard ratio are shown in Table 3: age (50-69 years, 2 points; ≥70 years, 3 points), underlying dementia (1 point), chronic renal failure (1 point), dyspnea (1 point), mental disturbance (1 point), ALC <1000/mm^3^ (1 point). We determined the COVID-19 prognosis score (COPS) based on the risk scores obtained for each patient and summing the respective scores of the 6 parameters. The total COPS ranged between 0 and 8.

**Figure 3:**
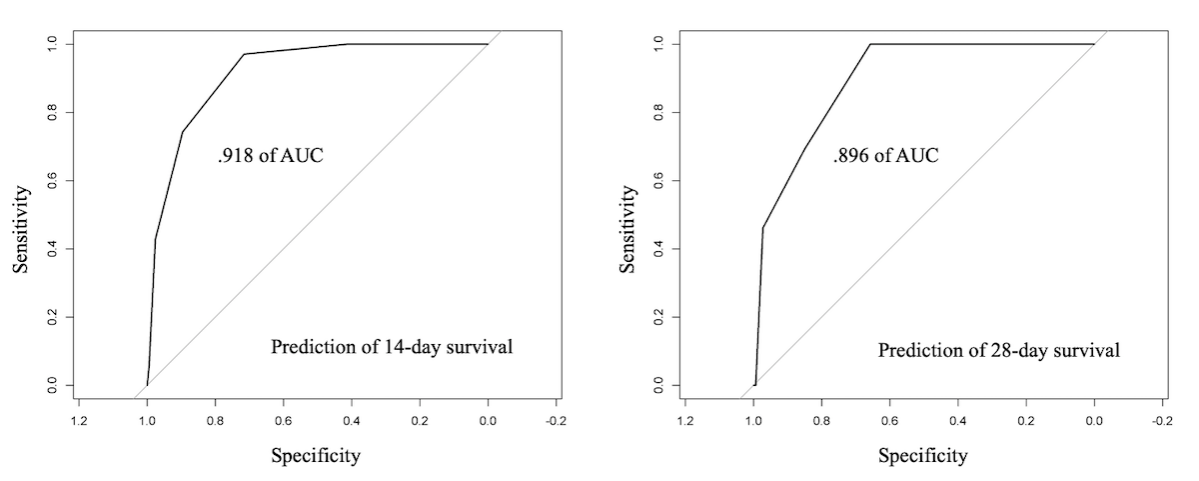
Receiver operating characteristics curve analysis of the newly developed COVID-19 prognosis score system in the validation cohort. A receiver operating characteristic curve analysis achieved an area under the curve value of (A) 0.918 (95% CI, 0.91-0.927) for 14-day survival and (B) 0.896 (95% CI 0.872-0.911) for 28-day survival.

**Figure 4:**
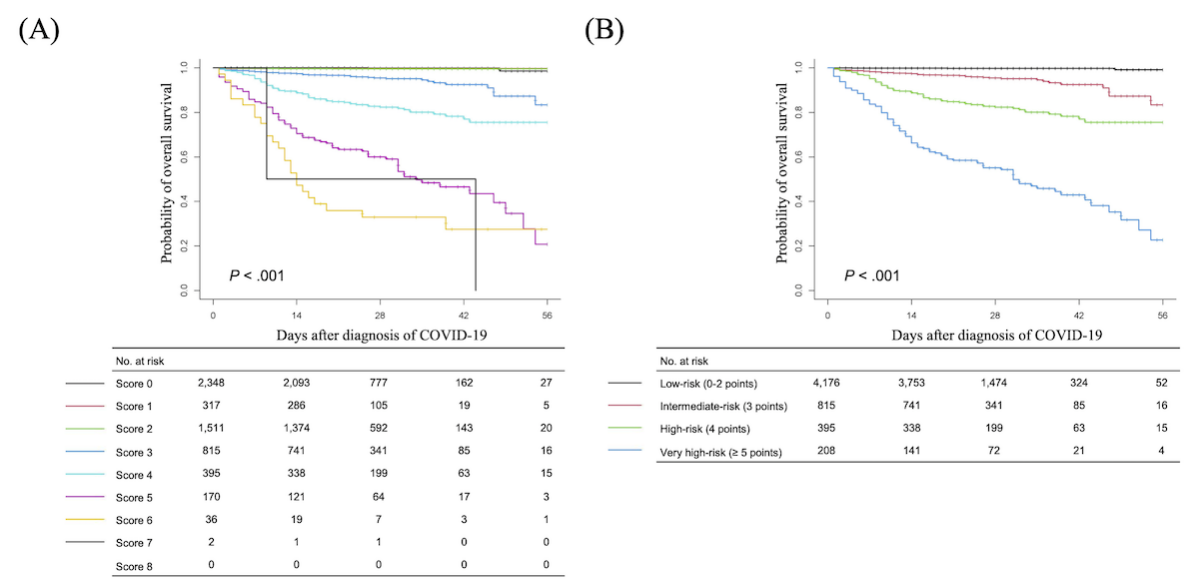
Probability of overall survival in patients with COVID-19 according to (A) each score and (B) the final COVID-19 prognosis score system.

We explored the clinical prediction score in the validation cohort using the AUROC curve analysis, which resulted in an AUROC value of 0.918 (95% CI 0.91-0.927) for the 14-day overall survival rate and 0.896 (95% CI 0.872-0.911) for the 28-day overall survival rate, indicating a reliable discrimination through the COPS system (Figure 3). Thereafter, we applied the scoring system to the total cohort, which resulted in a score range of 0-7 points (Figure 4A). This scoring system discriminately classified the patients into 8 groups. The 28-day overall survival rates were predicted as 99.9% (95% CI 99.7-100) in the 0-point group (n=2348), 99.7% (95% CI 99.1-100) in the 1-point group (n=317), 99.6% (95% CI 92.9-99.9) in the 2-point group (n=1511), 95.4% (95% CI 93.9-97.1) in the 3-point group (n=815), 82.3% (95% CI 78.5-86.4) in the 4-point group (n=395), 60% (95% CI 39.2-52.8) in the 5-point group (n=170), 32.9% (95% CI 20.6-52.7) in the 6-point group (n=36), and 50% (95% CI 12.5-100) in the 7-point group (n=2) (*P*<.001; Figure 4A).

We then determined the risk groups based on the final COPS system; these included low-risk (0-2 points, n=4167), intermediate-risk (3 points, n=774), high-risk (4 points, n=321), and very-high risk (≥5 points, n=98) groups. The 28-day overall survival rates for these groups were as follows: low-risk group, 99.8% (95% CI 99.6-99.9); intermediate-risk group, 95.4% (95% CI 93.9-97.1); high-risk group, 82.3% (95% CI 78.5-86.4); and very-high risk group, 55.1% (95% CI 48.5-62.5) (*P*<.001; Figure 4B). The developed COPS calculator is available online [17].

Furthermore, a significant increase in the length of hospital stay was observed as the risk group advanced: low-risk group, mean 25.4 (SD 10.4) days; intermediate-risk group, mean 27.2 (SD 10.9) days; and high-risk or very high-risk groups, mean 30.8 (SD 11.9) days (*P*<.001).

**Table 3:** The final scoring model in the development cohort. All values presented in the table represent data collected at the time of initial COVID-19 diagnosis.

Factor			Adjusted hazard ratio (95% CI)^a^		*P* value^a^		Log_e_ value of hazard ratio		Final score
**Age (years)**									
	<50		1 (reference)		N/A^b^		0		0
	50-69		6.7 (1.09-43.93)		.047		1.831		2
	≥70		26.03 (4.26-169.8)		<.001		3.186		3
**Sex**									
	Female		1 (reference)		N/A		N/A		N/A
	Male		1.35 (0.85-2.15)		.3		N/A		N/A
**Comorbidity**									
	**Hypertension**								
		No		1 (reference)		N/A		N/A	N/A
		Yes		1.21 (.75-1.94)		.48		N/A	N/A
	**Diabetes**								
		No		1 (reference)		N/A		N/A	N/A
		Yes		1.71 (1.08-2.7)		.61		N/A	N/A
	**Dementia**								
		No		1 (reference)		N/A		0	0
		Yes		3.92 (2.33-6.61)		<.001		1.35	1
	**Chronic cardiac disease^c^**								
		No		1 (reference)		N/A		N/A	N/A
		Yes		1.15 (.61-2.15)		.57		N/A	N/A
	**Cancer in active treatment**								
		No		1 (reference)		N/A		N/A	N/A
		Yes		1.83 (.72-4.71)		.30		N/A	N/A
	**Chronic pulmonary disease^d^**								
		No		1 (reference)		N/A		N/A	N/A
		Yes		1.76 (.857-3.625)		.25		N/A	N/A
	**Chronic renal failure**								
		No		1 (reference)		N/A		0	0
		Yes		3.48 (1.39-8.85)		.045		1.205	1
**Symptoms**									
	**Dyspnea**								
		No		1 (reference)		N/A		0	0
		Yes		2.31 (1.44-3.69)		.006		.821	1
	**Fatigue**								
		No		1 (reference)		N/A		N/A	N/A
		Yes		.69 (.3-1.57)		.38		N/A	N/A
	**Mental disturbance**								
		No		1 (reference)		N/A		0	0
		Yes		4.04 (1.72-9.53)		.04		1.268	1
**Systolic blood pressure (mmHg)**									
	<140		1 (reference)		N/A		N/A		N/A
	≥140		.93 (.57-1.54)		.59		N/A		N/A
**Diastolic blood pressure (mmHg)**									
	<80		1 (reference)		N/A		N/A		N/A
	≥80		1.03 (.63-1.68)		.63		N/A		N/A
**Heart rate (per min)**									
	<110		1 (reference)		N/A		N/A		N/A
	≥110		1.88 (.96-3.68)		.18		N/A		N/A
**Body temperature (°C)**									
	<38		1 (reference)		N/A		N/A		N/A
	≥38		.79 (.39-1.63)		.46		N/A		N/A
**Baseline hemogram**									
	**Hemoglobin (g/dL)**								
		≥12.5		1 (reference)		N/A		N/A	N/A
		<12.5		1.14 (.71-1.83)		.55		N/A	N/A
	**Absolute lymphocyte count (per mm^3^)**								
		≥1000		1 (reference)		N/A		0	0
		<1000		2.71 (1.66-4.43)		<.001		.982	1
	**Platelet count (per mm^3^)**								
		≥100,000		1 (reference)		N/A		N/A	N/A
		<100,000		1.68 (.77-3.7)		.34		N/A	N/A

^a^Based on 2000 bootstrap samples.

^b^Not applicable.

^c^Chronic cardiac disease was a composite variable including heart failure and cardiac disease.

^d^Chronic pulmonary disease was a composite variable including asthma and chronic obstructive lung disease.

## Discussion

In this study, we developed a new scoring system (COPS) to predict the mortality of patients with COVID-19 by using nationwide data of 5594 patients with COVID-19. The COPS system comprises basic demographics, initial symptoms, vital signs, and hemogram results at diagnosis. The risk score was stratified into four risk groups: low-risk, intermediate-risk, high-risk, and very high-risk groups associated with 28-day overall survival rate probabilities of 99.8%, 95.4%, 82.3%, and 55.1%, respectively. The AUROC curve analysis indicated that the prediction ability of the COPS system was excellent in the validation cohort.

Among the comorbidities identified in this study, dementia had the highest impact on mortality (adjusted hazard ratio 3.92) followed by chronic renal failure (adjusted hazard ratio 3.48). Moreover, the prognosis was poor when mental disturbance (adjusted hazard ratio 4.04) was noted at the time of diagnosis or when the patient had underlying dementia (adjusted hazard ratio 3.92). These two factors are related to the presence of SARS-CoV-2 infection clusters in nursing homes or long-term care facilities between February and April 2020 in Korea. Therefore, there is a need to establish screening and infection control strategies for long-term health care facilities [18-21].

In this study, an ALC of <1000/mm^3^ was found to affect the survival rate of patients with COVID-19. ALC has been used as a prognostic factor for common respiratory viruses, including respiratory syncytial virus or other viral reactivation in immunocompromised hosts [22]. A recent meta-analysis reported that lymphopenia on admission was associated with poor outcomes in patients with COVID-19 [23]. Further immunological studies on patients with COVID-19 are needed to elucidate the mechanism of lymphopenia and T cell reactivation, as well as cytokines [24].

Regarding disease severity, approximately 87% of patients did not need oxygen supplementation. Approximately 10% of the patients required oxygen supplementation; of these, 25% received oxygen via a simple mask or mechanical ventilators. From the perspective of a national strategy for new infectious disease crisis, it is important to determine the proportion of critically ill patients and the length of hospital stay according to disease severity in order to prepare medical resources, such as critical care beds. This study found that the hospitalization period was significantly longer among survivors than among nonsurvivors. Moreover, among the survivors, the length of hospital stay was directly associated with disease severity.

In this cohort, the infection fatality rate after COVID-19 diagnosis was 4.18% (234/5594). However, until April 30, 2020, the cumulative number of confirmed COVID-19 cases in Korea was 10,765 with 247 deaths, representing an actual mortality rate of 2.29% during the same period [25]. As of November 6, 2020, the total cumulative number of confirmed COVID-19 cases and deaths were 27,195 and 476, respectively, representing an infection fatality rate of 1.75% in Korea [25]. This disparity in mortality rates can be attributed to the fact that not all data were reported at the time this cohort was released. However, considering the large number of patients included in this cohort and collection of most deceased cases, the findings of this study are still meaningful and carry little statistical bias. In addition, remdesivir was not available in Korea during the study period, and infectious disease prevention and control measures were less established in the early phase of the COVID-19 pandemic. Thus, several mass infection episodes might have caused the relatively high mortality rate during the early phase of the pandemic in Korea.

Several studies have attempted to determine predictive factors for severe or fatal COVID-19 cases. A study on risk factors for fatal COVID-19 cases performed in China proposed a scoring system comprising age, procalcitonin, aspartate aminotransferase level, coronary heart disease, and cerebrovascular disease; this system was developed using data of 1590 inpatients with COVID-19 collected until January 2020. The nomogram showed discriminatory power with a C-index of 0.91 to predict survival [13]. Another study analyzed the risk of intensive care unit admissions and deaths among 4997 individuals under investigation at an academic hospital in New York. The study used age, heart rate, procalcitonin, lactate dehydrogenase, heart failure, and chronic obstructive pulmonary disease to predict care in intensive care units and deaths, yielding an accuracy of 0.74 and 0.83, respectively [26]. In the United Kingdom, a similar study was conducted on 17 million individuals included in the OpenSAFELY database, a near–real-time primary care patient record, which pseudonymously identified 10,926 COVID-19–related deaths. The study found male sex, older age and deprivation, diabetes, and severe asthma as significant risk factors for death [27]. However, the study did not analyze survival and death among patients with a confirmed COVID-19 diagnosis. In addition to studies attempting to predict death in patients with COVID-19, other studies have focused on severity index to predict severe or critical cases [10,28-31]. More recently, a machine learning–based warning system for mortality risk prediction of patients with COVID-19 was reported, and timely risk stratification using multiple laboratory and clinical factors was improved [32].

Compared with the abovementioned studies, our study has a number of strengths. First, the risk factors for death were analyzed using a nationwide cohort comprising a large number of patients with COVID-19, which resulted in a scoring system that can be widely used for triaging laboratory-confirmed COVID-19 cases. Second, the COPS system was developed using easily accessible information, such as age, underlying disease, dyspnea, mental disturbance, and hemogram parameters. We believe that if a scoring system that uses only simple laboratory tests (eg, hemogram parameters) can be developed and still show good predictability, it will prove to be more cost-effective than systems including other biomarkers such as procalcitonin or cytokine levels. Third, the discriminatory power of our system for predicting death probability was excellent. Finally, this study further analyzed the length of hospital stay according to disease severity, which may assist in preparing medical resources based on patient classification. However, this study is limited by the lack of external verification for our scoring model. Therefore, the current model of overall survival for the diagnosis of COVID-19 would need to be validated in a future cohort.

In conclusion, our study provides a simple scoring system based on information collected at diagnosis for predicting mortality among patients with COVID-19 in a timely manner. Early triaging of patients with COVID-19 using the COPS system can provide new insights for risk-adaptive strategies and optimize the use of medical resources.

## Abbreviations

ALC: absolute lymphocyte count
AUROC: area under the receiver operating characteristic curve
COPS: COVID-19 prognosis score
ECMO: extracorporeal membrane oxygenation
KDCA: Korea Disease Control and Prevention Agency
